# Development of food photographs for use with children aged 18 months to 16 years: Comparison against weighed food diaries – The Young Person’s Food Atlas (UK)

**DOI:** 10.1371/journal.pone.0169084

**Published:** 2017-02-15

**Authors:** Emma Foster, Adrian Hawkins, Karen L. Barton, Elaine Stamp, John N. S. Matthews, Ashley J. Adamson

**Affiliations:** 1 Human Nutrition Research Centre, Institute of Health and Society, Newcastle University, Newcastle, United Kingdom; 2 Division of Food and Drink, School of Science, Engineering & Technology Abertay University, Dundee, United Kingdom; 3 School of Mathematics and Statistics, Newcastle University, Newcastle, United Kingdom; Old Dominion University, UNITED STATES

## Abstract

Traditional dietary assessment methods, used in the UK, such as weighed food diaries impose a large participant burden, often resulting in difficulty recruiting representative samples and underreporting of energy intakes. One approach to reducing the burden placed on the participant is to use portion size assessment tools to obtain an estimate of the amount of food consumed, removing the need to weigh all foods. An age range specific food atlas was developed for use in assessing children’s dietary intakes. The foods selected and portion sizes depicted were derived from intakes recorded during the UK National Diet and Nutrition Surveys of children aged 1.5 to 16 years. Estimates of food portion sizes using the food atlas were compared against 4-day weighed intakes along with in-school / nursery observations, by the research team. Interviews were conducted with parents the day after completion of the diary, and for children aged 4 to 16 years, also with the child. Mean estimates of portion size consumed were within 7% of the weight of food recorded in the weighed food diary. The limits of agreement were wide indicating high variability of estimates at the individual level but the precision increased with increasing age. For children 11 years and over, agreement with weighed food diaries, was as good as that of their parents in terms of total weight of food consumed and of intake of energy and key nutrients. The age appropriate food photographs offer an alternative to weighed intakes for dietary assessment with children.

## Introduction

Accurate information on the dietary intake of children is essential to inform nutrition related health policies and evaluate interventions. Obtaining information on food intake presents many challenges. There tends to be a trade-off between the accuracy of a method and the burden it places on the study participant. More accurate methods such as duplicate diet and weighed food diaries are costly and more burdensome and may result in under-reporting of energy intake[1, 2], participant selection bias and recording bias[3] along with subconscious and / or intentional changes to diet to facilitate recording[4]. One method of overcoming some of these challenges is to reduce participant burden by using portion size assessment tools to obtain an estimate of the amount of food consumed. Portions size estimation tools have been developed using one or a combination of; two or three dimensional drawings[5], food models[5], digital images of foods and food receptacles[6–8], and digital graphics of shapes and mounds[6, 8] and have been used with varying degrees of success.

Estimation rather than weighing of portion size is associated with a loss of precision with both over- and under-estimation, however printed photographs of foods have been shown to increase the accuracy of food portion estimation compared with unaided estimates[9, 10], and to result in an increase in accuracy of nutrient intake information[9–11]. In the UK, the Photographic Atlas of Food Portion Sizes[12] has been developed for use with adults however, no similar tool exists for use with children. Frobisher and Maxwell (2003) and Foster *et al*. (2006) found that errors in portion size estimation made by children[13, 14] using the adult food photographs[12] were much greater than those observed when adults used the photographs. The authors concluded that the tool was unsuitable for use with children. This led to the question—Can children accurately estimate food portion sizes when using age-specific portion size estimation tools?

A pilot study was conducted in 2003 to assess the effect of perception, conceptualisation and memory on children’s ability to estimate the portion size of foods. This study has been reported previously[15] and is described briefly here. Three age-specific portion size assessment tools were developed: food photographs, food models and an interactive portion size assessment system (IPSAS). A feeding study was conducted to test the validity of children’s estimates of portion size using the 3 tools. Children (n = 201) aged 4–16 years were provided with pre-weighed portions of 22 foods to consume in school. The amount of any food leftover was weighed and recorded. Children estimated the amount of food served and leftover the following day using one of the tools. The children were given the same food on several occasions and estimates of portion size were made for each food using each tool seperately. A total of 9843 estimates of portion size were made. Two of the tools (food photographs and IPSAS) showed good potential for estimating portion sizes of foods consumed by children aged 4 to 16 years[15].

The pilot study included a narrow range of foods and children were fed in school, away from their normal routine. This paper describes the further development of the age appropriate photographs and testing in a real life-setting, during a nutritionist administered interview following the completion of a 4-day weighed food diary. Futher development and testing of IPSAS is described by Foster *et al* (2014)[16].

## Aims

The aims of the study were to: further develop the age appropriate food photographs into a comprehensive tool for total dietary assessment with children and to conduct a comparison of the food photographs against weighed intakes. To maximise utility, three separate versions of the Young Person’s Food Atlas (YPFA) were developed for use with children of pre-school age (18 months-4 years), primary school age (4–11 years) and secondary school age (11–16 years).

## Methods

### Selecting foods to include in the Atlases

The original food photographs used in the pilot study were supplemented by additional images in order to cover the top 100 foods based on frequency of consumption, weight of food consumed and contribution to energy intake of foods consumed by children in the National Diet and Nutrition Surveys (NDNS) of children aged 1.5–4.5 years, and young people aged 4–18 years. There was significant overlap and the union of the top 100 foods, using these 3 criteria, resulted in a total of 104 foods[17, 18]. Taking into account the use of food photographs for estimating the portion size of equivalent foods (e.g slices of ham used in the estimation of other types of sliced meat) the YPFA is suitable for the estimation of portion size of approximately 85% of the foods consumed by children in the NDNS on a weight basis[18]. The criteria for inclusion was chosen given that beyond this there would have been limited return, for example, the 100th food in terms of weight consumed accounted for only 0.1% of the total weight of food consumed and 0.2% of total energy intake, therefore adding further foods would add little additional coverage. It is acknowledged however that in order to investigate specific micronutrients which are present in large amounts in infrequently consumed foods e.g. n-3 fatty acids in mackerel or vitamin A in liver it may be necessary to supplement the atlas with images of these foods.

### Presentation of foods

Portion size photographs were developed for estimation of amount served and amount leftover. This decision was made because children often leave a proportion of the foods which they are served and therefore, in many instances, asking for an estimate of amount consumed would require a child (or their parent) to conceptualise an amount of food they had never actually seen (as they would see the amount served, the amount leftover but not the amount consumed)[5].

**Fig 1:**
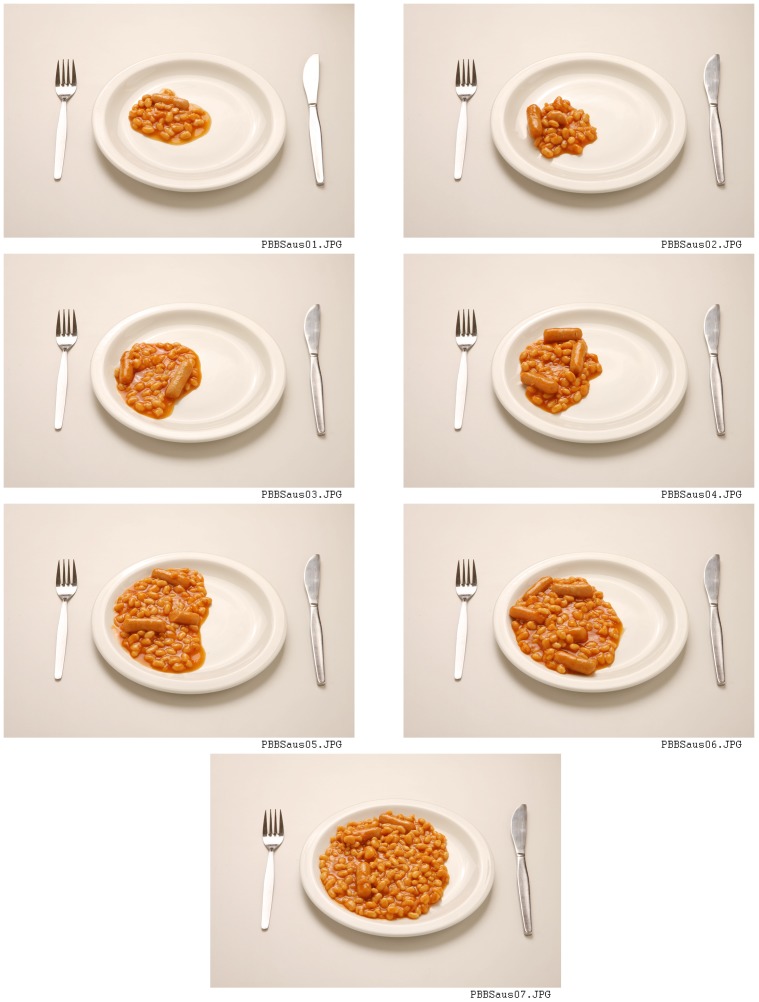
Example page from food atlas– 7 portion photographs for estimates of amount served and leftover.

**Fig 2:**
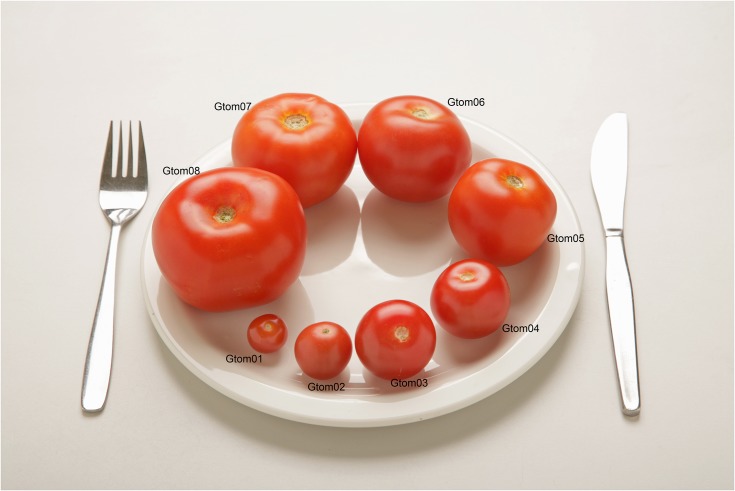
Example page from food atlas—guide photograph.

The food photographs are displayed in two formats:

1. 7 portions for estimation of amount served and 7 portions for estimation of amount leftover for foods which are not usually presented in predetermined amounts, for example baked beans or broccoli (Fig 1).
2. Guide photographs for foods, which are usually presented in predetermined amounts, such as bread rolls, where a range of commonly served portion sizes are displayed in one photograph (Fig 2). For these foods there are no photographs for estimation of amount leftover.

For a small proportion of foods there is no suitable photograph (e.g. foods which are consumed infrequently). For these foods a description of the portion size was collected using household measures, brand and pack information, or comparison to a standard sized object.

For the 7 portions, for estimation of amount served, weights from the 5^th^ to the 95^th^ centile of weight served during the NDNS[17, 18] were calculated as equal increments on a log scale for each age group. Seven weights from the 5^th^ centile (the smallest as served portion) to the smallest presentable portion were used for estimation of the amount of any food leftover. The portion sizes are presented on a log scale as sensory systems (such as vision) respond in a logarithmic fashion to objects in the external world[19]. This means that if the increments between a range of photographs are of equal gram weight (e.g. 10g increments) it is more difficult to detect the difference between the two largest portion photos e.g. 60g and 70g (a difference of +17%) than between the smallest two e.g. 10g and 20g (a difference of +100%). On a log scale the portions increase proportionately so, in the case of a range of portions from 10g to 70g there will be a difference of +38% between each photograph.

### Design of the Young Person’s Food Atlas

The 2055 photographs of 104 foods were organised in a printed food atlas by food category for example: cereals and cereal products; meat and meat products; vegetables; etc. The atlas is 206 pages long and is designed for use by a researcher during a dietary interview for a 24hr recall or estimated weight food diary. An A3 life size image of the crockery used in the photographs is included at the front of the atlas. The researcher asks the child or their parent to estimate the amount of food served and, if appropriate, the amount of any food leftover. The 7 portions for estimation of amount served are presented on one side of A4 with the smaller portions for estimation of amount leftover printed overleaf (Fig 1). The guide photographs are printed landscape covering the full A4 page (Fig 2). Three versions of the atlas were created depicting age-appropriate portion sizes for pre-school, primary and secondary school children.

### Study population

Children aged 1.5 to 16 years were recruited from Newcastle upon Tyne, UK, and the surrounding area. Schools and nurseries were asked to participate based on free school meal index as a proxy for level of deprivation in order to provide a range of catchment areas. 10 nurseries were approached and 7 agreed, 18 schools were approached and 9 agreed. A recruitment letter was sent to parents of all children attending each school or nursery. Written parental consent and, for children of secondary school age, child assent were sought for participation in the study. Parents of pre-school children cared for at home were recruited via local mother and toddler groups and posters within the community. Children were selected for inclusion in the study using a measure of deprivation based on postcode known as the Index of Multiple Deprivation (IMD)[20]. This study was conducted according to the guidelines laid down in the Declaration of Helsinki and all procedures involving human subjects were approved by the Newcastle University Ethics Committee.

### Sample size

The sample size calculations are based on the method described by Bland and Altman (1986)[21] and allow estimation of the limits of agreement to be determined to within 10% of the width of the region of agreement for each age group. The target was to complete assessments in 300 children (100 in each of 3 age-groups—preschool, primary school age and secondary school age children) with a recruitment target of 360 children (120 in each age-group) to allow for attrition.

### Comparison of YPFA estimates against weighed food diaries

#### Weighed food diaries

Parents were provided with calibrated digital food scales and asked to keep a 4-day weighed record of their child’s food intake covering two weekend days and two weekdays. Both weekend days were included as research suggests that intake on Saturday and Sunday vary greatly from each other and that to obtain estimates that are representative of usual food and nutrient intakes both weekend days and a selection of week days should be measured[22]. Home intake was recorded by the parent / carer, with assistance from the child themselves for children aged 11 years and over. To ensure the accuracy of data collected for foods consumed at school and nursery, the research team weighed each food served and any plate waste. In nursery school it was possible to weigh the food as it was served onto the individuals’ plate. In primary schools duplicate samples of each meal were used due to practical issues around weighing foods served in a busy school canteen. As it was felt a researcher collecting the samples may impact on the amount served, children were instructed (out of view of the catering staff) which foods to select. The tray containing the foods was then taken from the child (who was free to then select their own meal) and the foods weighed using calibrated kitchen scales accurate to 1g by trained research staff. Each individual child’s plate waste was also weighed.

#### Interviews using the Young Person’s Food Atlas

Interviews, to obtain an estimate of portion size for each food, took place the day following completion of the food diary (day 5). The interviewer used the weighed food diary as a record of foods consumed working through the diary in chronological order asking for an estimate of amount served and leftover for each food using the age-appropriate YPFA. For pre-school children, interviews took place with parents in their home. For children of school-age both child and parent were interviewed. Children were interviewed in school and their parents at home to avoid possible contamination between interviewees. In research and nutrition surveillance portion size estimation aids could be used during an interview for a 24hr dietary recall or during an interview for an estimated weight food diary where respondents could be asked to estimate the portion size of foods consumed several days previously. The study design allowed us to examine how estimates made using the YPFA differed as the time between consuming the item and estimating the amount increased.

### Statistical analysis

The results are presented as ratios of estimated to actual weight of food or drink. This allows data from foods for which the usual portion sizes differ widely, such as butter and mashed potato, to be combined in a meaningful way. While amalgamation of such a broad range of foods undoubtedly hid some important sources of variation, it allowed succinct, broad comparisons of the performance of the YPFA between different groups of subjects.

The analysis was performed on the logarithms of the weights, a methodology which also makes the distribution of weights for some foods much closer to Normal. If the weight of a given food is *W* and this is estimated using the photographs as *E*, as *E* and *W* purport to measure the same quantity, the method comparison technique of Bland and Altman (1986)[21] was applied to the log *E* and log *W* values. The 95% limits of agreement were computed as *d* +/- 1.96*s*, where *d* and *s* are the mean and standard deviation of the differences log *E—*log *W*. These are reported as the antilogarithm of *d*, i.e. the geometric mean of the *E*/*W* ratios and the antilogarithms of the limits of agreement, which provide an interval within which 95% of the ratios lie.

Analyses examine the agreement between the weighed food diaries and estimates made using the YPFA for parents and children, for the top 20 foods in terms of frequency of consumption and also by length of time between consuming and estimating the portion size of the food.

## Results

### Recruitment and completion statistics

The target was to include 100 children in each age group (pre-school, primary and secondary school) and for these children to be representative of the national population in terms of indicators of deprivation by Index of Multiple Deprivation (IMD)[20] based on home postcode and ethnicity. In total 411 consents were obtained, from which 379 children were selected for inclusion in the study based on gender and IMD. 83% percent of children completed the 4-day food diary and interview. Attrition of participants was observed to a lesser degree in pre-school (88% completing) and primary school children (85%) than secondary school age children (75%). The completion rates are high considering the large participant burden of 4 days of weighed intake. Weighed intakes often result in high attrition rates and or varying degrees of data completion, especially when teenagers are the participants. The UK National Diet and Nutrition Survey (NDNS): Young people aged 4 to 18 years[18] found a greater proportion of non-responders in the 15–18 year old age group (56% response rate) compared with the 4–6 year old age group (66% response rate).

The average IMD for pre-school children was 22.0 (range 2.8–76.1), close to the UK national average of 21.6 (IMD ranges from 0–65 and the higher the IMD the more deprived the location). For primary and secondary school children our sample was of higher deprivation on average than the national average (34.6 and 27.0 respectively). Children from minority ethnic groups comprised 6.1% of the sample compared with a national average of 7.9% of the population (Table 1).

**Table 1:** Recruitment and demographics of participants completing the study.

Age group	No. consenting	No. Included	No. (%) completing		%Ethnic Minority	IMD*Mean (Range)
**Preschool**	**123**	**120**	**105**	**(88%)**	**8.6**	**22.0 (2.8–66.1)**
**Primary**	**160**	**131**	**112**	**(85%)**	**5.4**	**34.6 (3.6–76.1)**
**Secondary**	**128**	**128**	**96**	**(75%)**	**4.2**	**27.0 (4.0–76.1)**
**Total**	**411**	**379**	**313**	**(83%)**	**6.1**	**28.0 (2.8–76.1)**

* IMD = Index of Multiple Deprivation

### Agreement of portion size using YPFA with weighed food diaries

In total over 24,000 individual estimates of portion size were obtained using the YPFA. Parents of pre-school children were found to under-estimate the amount served on average by 5% and over-estimate the amount consumed by 7% (Table 2). Estimates of portion size made by Primary school age children showed good agreement with weighed food diaries for their estimates of the portion size served (+1% on average) but they tended to over-estimate the amount of food consumed (+7%), perhaps indicating issues in reporting of food leftover in this age-group. By contrast parents of primary school children tended to under-estimate the amount consumed compared with weighed food diaries (-5%). The mean ratio and limits of agreement for the Secondary school children were very similar to their parents. Both parents and children tended to slightly under-estimate the amount consumed compared with weighed food diaries (-5% and -4% respectively).

**Table 2:** Agreement of estimates of portion size using YPFA with weighed food diaries—by age group and respondent.

Age group	Respondent	Ratio of estimated to actual weight	n =	Mean ratio*	Limits of Agreement		% within	
					Lower	Upper	50%	10%
**Preschool**	**Parent**	**Weight served**	5656	0.95	0.26	3.52	67	19
		**Weight consumed**	5656	1.07	0.25	4.57	62	18
**Primary**	**Child**	**Weight served**	4387	1.01	0.26	3.87	64	19
		**Weight consumed**	4387	1.07	0.25	4.65	61	18
	**Parent**	**Weight served**	4326	0.90	0.26	3.18	68	19
		**Weight consumed**	4326	0.95	0.25	3.67	66	19
**Secondary**	**Child**	**Weight served**	4915	0.94	0.26	3.44	68	21
		**Weight consumed**	4915	0.96	0.25	3.67	67	21
	**Parent**	**Weight served**	4730	0.92	0.26	3.22	71	20
		**Weight consumed**	4730	0.95	0.26	3.52	70	21

* The ratio is the estimated weight using the YPFA divided by the weighed weight recorded in the food diary. Therefore a figure of 1 would indicate exact agreement, a value of >1 indicates overestimation of portion size using the YPFA and a value of <1 indicates underestimation using the YPFA.

**Fig 3:**
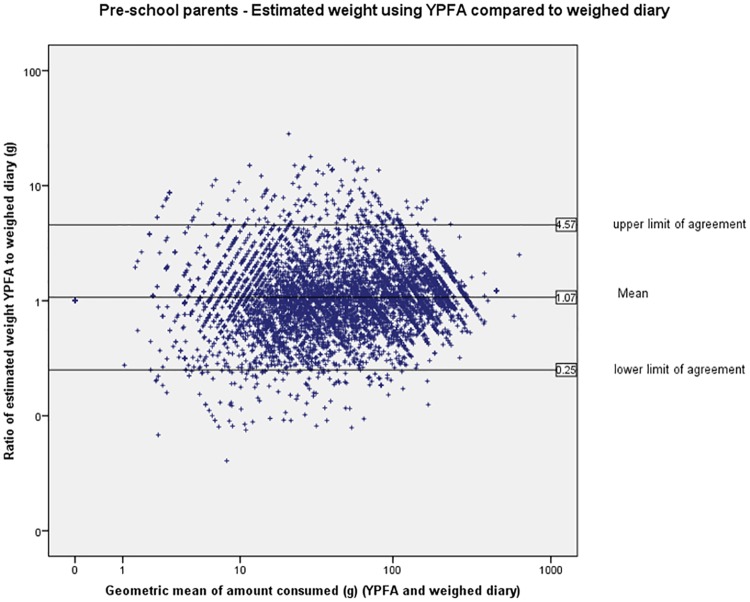
Pre-school parents—Bland-Altman plot showing estimated weight using YPFA compared to weighed diary. Note the data points show the individual estimate of food weight using the YPFA divided by the weight recorded for that food in the weighed diary. A value >1 indicates overestimation of portion size using the YPFA and a value of <1 indicates underestimation using the YPFA. If the mean line is at 1 this would indicate that there is no bias of one method relative to the other, and the degree of agreement at the group level is indicated by the limits of agreement, within which 95% of observations lie.

**Fig 4:**
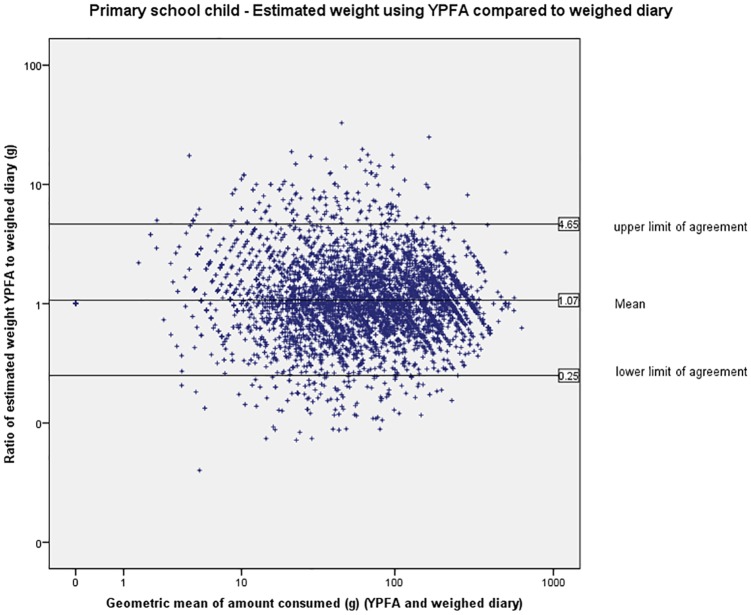
Primary school children—Bland-Altman plot showing estimated weight using YPFA compared to weighed diary.

**Fig 5:**
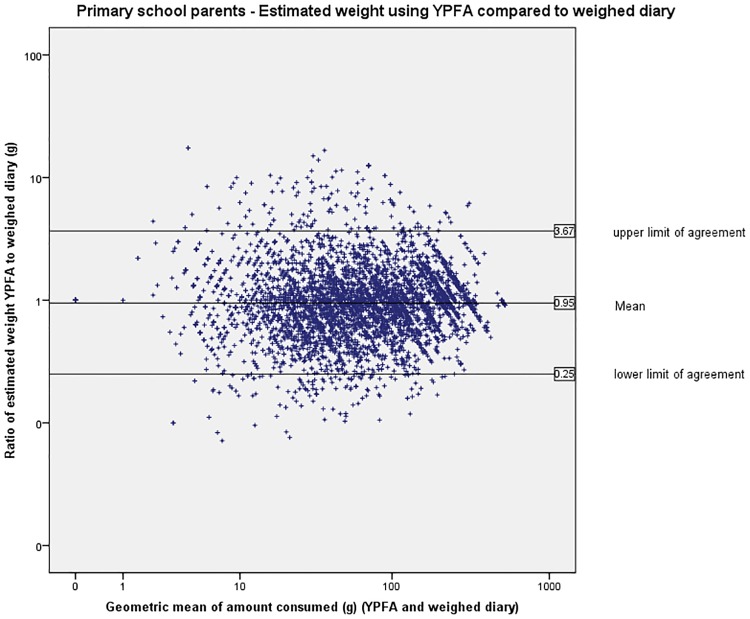
Primary school parents—Bland-Altman plot showing estimated weight using YPFA compared to weighed diary.

**Fig 6:**
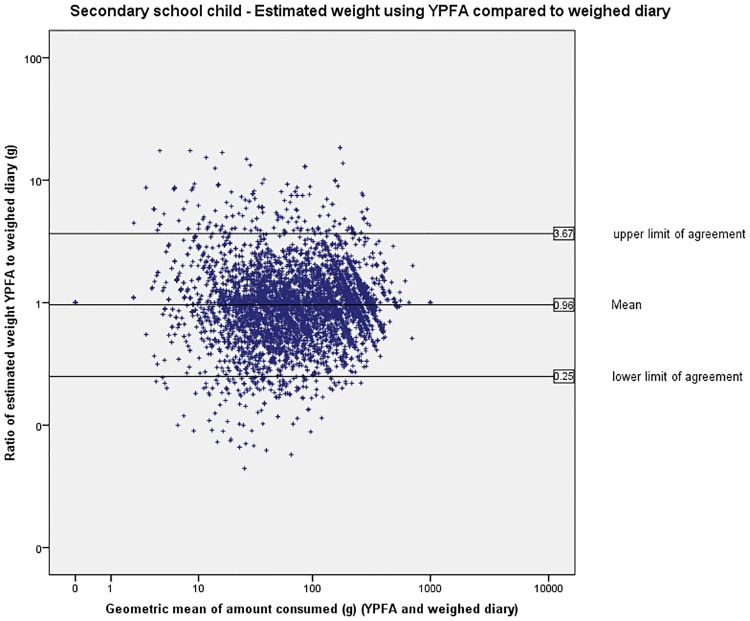
Secondary school children—Bland-Altman plot showing estimated weight using YPFA compared to weighed diary.

**Fig 7:**
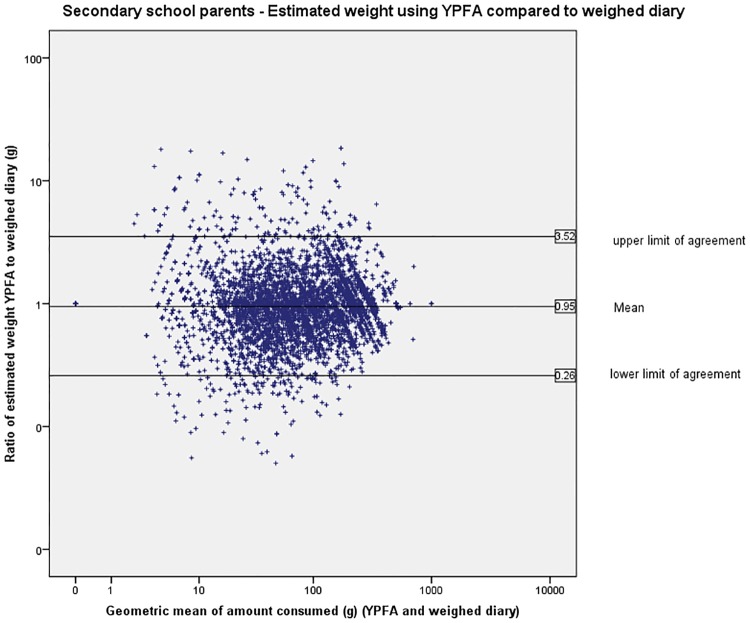
Secondary school parents—Bland-Altman plot showing estimated weight using YPFA compared to weighed diary.

**Fig 8:**
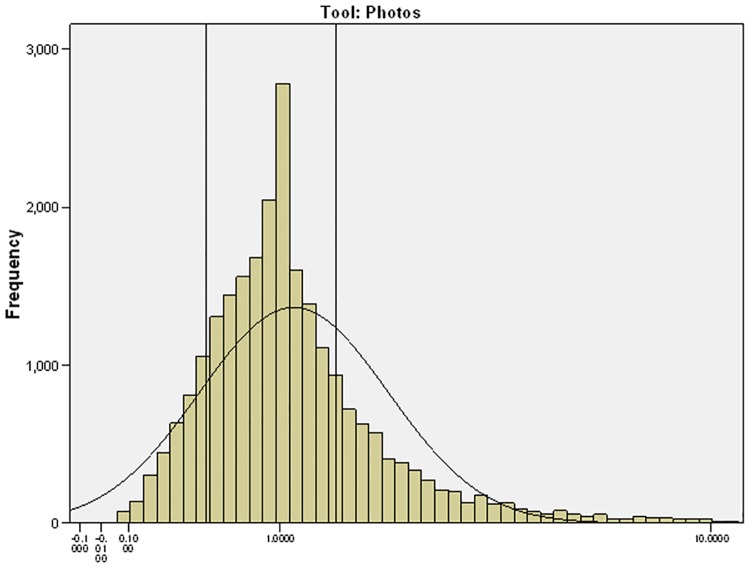
Histogram displaying the range in the ratio of estimated weight to actual weight for estimates made using YPFA of the amount of food consumed (50% of the total number of estimates lie between the two vertical lines on the graph).

The limits of agreement (LOA) are wide indicating large variation in individual estimates of portion size (Figs 3 to 7). The LOA for parents of pre-school children were from an under-estimate of 75% of the weight reported in the weighed diary to an over-estimate of 357%. For estimates made by the primary school children the limits are even wider from an under-estimate of 75% to an over-estimate of 365%. The LOAs do improve as the age of the child increases and are narrowest for the secondary school children’s parents where they range from an under-estimate of 75% to an over-estimate of 252% however, this still represents large individual variation. Fig 8 shows the distribution of the ratio of estimated weight to actual weight consumed for estimates made using YPFA. The majority of estimates are close to 1 (demonstrating very good agreement) but the limits of agreement are skewed by a small number of wildly inaccurate estimates (Fig 3). The percentage of estimates correct to within 50% of the actual weight ranged from 61% for primary school children’s estimates of the amount consumed to 71% for parents of secondary school children’s estimates of amount served. Percentage of estimates correct to within 10% of the actual weight ranged from 17.7% for primary school children’s estimates of the amount consumed to 21% of secondary school children’s estimates of the amount served (Table 2).

A suitable photograph was available for estimation of 91% of foods consumed, interestingly the proportion was slightly higher for participants from an ethnic minority background. For the remaining 9% of foods a description of amount consumed was collected. Of these 6% were items which are in predetermined amounts (e.g. biscuits and crisps; the UK name for potato chips) while 3% were items not in predetermined amounts (e.g. blueberries and stuffing). Agreement was better for foods in predetermined amounts which were over-estimated by 3% on average compared with 13% over-estimation for foods not in predetermined amounts.

Analysis of the mean ratio and limits of agreement by day recalled revealed no clear pattern for agreement to increase as the time between consuming the food and estimating portion size decreased, suggesting the YPFA can be used in conjunction with a 4-day estimated food diary as well as for 24hr recalls (Table 3).

**Table 3:** Agreement of estimates of portion size using YPFA with weighed food diaries for day 1 of the food diary compared with day 4.

Participant	Day	n = *	Mean ratio†	Limits of Agreement	
				Lower	Upper
**All**	**1**	6139	1.02	0.25	4.11
	**2**	6372	0.99	0.25	3.98
	**3**	5889	1.00	0.25	3.98
	**4**	5482	1.01	0.24	4.15
**Child**	**1**	2393	1.04	0.25	4.25
	**2**	2497	0.99	0.24	4.02
	**3**	2269	1.01	0.25	3.98
	**4**	2090	1.02	0.24	4.28
**Parent**	**1**	3746	1.00	0.25	3.97
	**2**	3875	0.99	0.25	3.93
	**3**	3620	0.99	0.25	3.95
	**4**	3392	1.00	0.25	4.04

* The difference in numbers between Table 3 and Table 2 is 132 –due to a few entries not having the day of recall identified.

^†^The ratio is the estimated weight using the YPFA divided by the weighed weight recorded in the food diary. Therefore a figure of 1 would indicate exact agreement, a value of >1 indicates overestimation of portion size using the YPFA and a value of <1 indicates underestimation using the YPFA.

### Variation by food type

There was a great deal of variation in agreement between estimates made using the YPFA and the weighed food diaries by food type (Table 4). This was investigated for the 20 most frequently reported items. The foods and drinks which showed the best agreement were biscuits which were accurately estimated on average, chocolate bars which were over-estimated by 1% on average, and yoghurts which were under-estimated by 1% on average, in comparison with the weighed food diaries. There was no clear pattern for estimation to vary according to the morphology of the food. Those foods for which agreement was poorest included milk on cereal which was over-estimated by 42% on average, compared with the weighed food diaries and over-estimated by almost 140% by children. The wide limits of agreement for both parents and children demonstrate that the estimation of milk on cereal was particularly challenging. The over-estimation seen for bananas and apples may be due to errors in reporting whether the weight recorded in the diary included the peel or core. The foods which were under-estimated to the greatest degree in comparison with the weighed food diaries include Rice Krispies, chips (chips are the UK name for french fries) and carrots which were under-estimated by 31%, 30% and 26% respectively. The limits of agreement are wide for all food types indicating large variation in agreement at the individual level.

**Table 4:** Agreement of estimates of portion size using YPFA with weighed food diaries—by food type.

Food Type	*All Participants*				*Parent*				*Child*			
	n =	Mean ratio*	Limits of Agreement		n =	Mean ratio*	Limits of Agreement		n =	Mean ratio*	Limits of Agreement	
			Lower	Upper			Lower	Upper			Lower	Upper
**Bananas**	286	1.98	0.66	5.93	193	2.13	0.69	6.61	93	1.69	0.65	4.41
**Milk on Cereal**	496	1.42	0.21	9.44	346	1.14	0.17	7.67	150	2.38	0.60	9.52
**Apples**	370	1.36	0.27	6.79	229	1.46	0.26	8.24	141	1.21	0.31	4.71
**Milk as a drink**	1017	1.16	0.36	3.72	766	1.23	0.39	3.89	251	0.99	0.32	3.06
**Hot drinks**	523	1.16	0.45	3.01	276	1.18	0.47	2.99	247	1.14	0.43	3.03
**Butter**	826	1.15	0.22	5.87	499	1.09	0.21	5.65	327	1.24	0.25	6.16
**Soft drinks**	3452	1.12	0.35	3.56	2075	1.17	0.36	3.84	1377	1.05	0.35	3.13
**Bottle drinks**	679	1.08	0.43	2.71	416	1.10	0.43	2.78	263	1.06	0.43	2.62
**Crisps (Chips, US)**	562	1.02	0.40	2.63	318	0.99	0.37	2.67	244	1.07	0.45	2.54
**Toast**	399	1.02	0.31	3.29	223	1.00	0.31	3.20	176	1.04	0.32	3.41
**Chocolate bars**	471	1.01	0.32	3.24	261	1.01	0.32	3.16	210	1.01	0.31	3.36
**Biscuits**	245	1.00	0.30	3.39	146	1.01	0.33	3.05	99	0.99	0.25	3.91
**Yoghurt**	310	0.99	0.33	3.00	190	0.95	0.32	2.81	120	1.06	0.34	3.31
**Hard Cheese**	341	0.97	0.24	4.04	225	0.94	0.24	3.66	116	1.05	0.23	4.83
**Sliced Bread**	839	0.97	0.33	2.81	505	1.00	0.35	2.86	334	0.92	0.31	2.71
**Bread rolls**	303	0.96	0.31	3.02	181	1.02	0.33	3.16	122	0.88	0.28	2.79
**Ham**	252	0.94	0.25	3.61	165	0.94	0.26	3.46	87	0.95	0.23	3.93
**Boiled Carrots**	280	0.74	0.20	2.77	176	0.73	0.19	2.76	104	0.74	0.20	2.81
**Chips (Fries, US)**	338	0.70	0.18	2.81	198	0.64	0.17	2.41	140	0.80	0.19	3.34
**Rice Krispie Type Cereals**	244	0.69	0.22	2.13	149	0.60	0.20	1.84	95	0.85	0.31	2.35

* The ratio is the estimated weight using the YPFA divided by the weighed weight recorded in the food diary. Therefore a figure of 1 would indicate exact agreement, a value of >1 indicates overestimation of portion size using the YPFA and a value of <1 indicates underestimation using the YPFA.

### Impact of errors on reported nutrient intake

The mean ratio given in Table 5 is the mean daily amount of energy or a given nutrient reported using YPFA divided by the amount reported in the corresponding weighed diary. Thus for each individual child’s food diary we have up to 2 estimates (parent and child). Using the YPFA there was a very slight over-estimate of 1% of the weight of food consumed with limits of agreement from an under-estimate of 36% to an over-estimate of 62%, 95.5% of the estimates were within 50% of the actual weight of food consumed recorded in the weighed food diary (Table 5).

**Table 5:** Mean ratio of daily energy and nutrient intakes reported by estimated intakes using YPFA to intakes reported by weighed intake.

	n = *	Mean ratio†	Limits of Agreement		% within 50%
			Lower	Upper	
**Ratio Wt of food**	537	1.01	0.64	1.62	95.5
**Ratio Energy**	537	0.97	0.60	1.58	96.3
**Ratio Protein**	537	0.95	0.54	1.68	92.7
**Ratio CHO**	537	0.98	0.61	1.57	96.3
**Ratio Fat**	537	0.95	0.53	1.73	90.9
**Ratio Iron**	537	0.90	0.52	1.55	95.2
**Ratio Vitamin C**	537	1.03	0.51	2.11	85.1

* The number of estimates relates to the number of completed estimated food diaries by parents and children.

^†^The mean ratio is the mean daily intake reported using the YPFA divided by the mean daily intake reported using the weighed food diary. Therefore, a figure of 1 would indicate exact agreement, a value of >1 indicates overestimation of intake using the YPFA and a value of <1 indicates underestimation using the YPFA.

In terms of mean daily energy intake YPFA under-estimated energy intake by 3% compared with the weighed food diary. The limits of agreement were from an under-estimate of 40% to an over-estimate of 58% of energy and 96.3% of the estimates lay within 50% of the energy intake reported in the weighed food diary. Intakes of protein and fat were under-estimated by 5% on average using the photographs (the limits of agreement (LOA) were from an under-estimate of 46% and 47% to an over-estimate of 68% and 73% for protein and fat respectively). Carbohydrate was under-estimated by 2% on average (LOA -39% to +57%), Iron intakes were under-estimated by 10% (LOA -48% to +55%) and Vitamin C intakes were very slightly over-estimated on average by 3% (LOA -49% to +111%).

## Discussion

Using the YPFA adults under-estimated their child’s portion size by 1% on average, with 19.2% of estimates within 10% of the actual amount consumed whereas children over-estimated portion size by 1% on average and 19.1% of estimates were within 10% of the actual amount consumed. These figures are comparable to the findings of Nelson *et al*. (1996) who found that adults, using photographs of adult food portion sizes of 26 foods, over-estimated amounts by an average of 11%[23]. In the US, in the formative development of ASA24, a computer based 24 hour recall for use with adults, Subar *et al*. (2010) found 14.8% of the estimates for 27 different foods were within 10% of actual amount consumed[8].

Foods which proved most challenging to estimate portion size using the food photographs include milk on cereal, apples and chips. Milk on cereal was over-estimated by 41% on average in comparison with weighed intakes and there was large variation in estimates as indicated by the wide limits of agreement. Milk on cereal was presented as a ratio of milk to cereal with leftovers presented as milk in a bowl on their own. This represents a very complex cognitive task. Chips were under-estimated by 29% on average using the food photographs compared with the weighed intakes. This large difference may be due, in part, to the degree of cooking of the chips as oven chips will lose weight due to cooking but with little change in size or shape (or indeed in, nutrient content). This will apply to other foods which differ in weight to a greater degree than they differ in size and appearance with cooking. Biscuits, yoghurt, chocolate bars and toast were the foods for which best the agreement was seen, possible due to their being less potential variation in portion size along with easy identification of packaged items.

Hernández *et al*. (2006) observed wide variation in the accuracy of estimates when analysis is carried out on individual foods and suggest that simply reporting the overall mean values may mask true errors in estimation[24]. Korkalo *et al*. (2012) found estimates of portion size made by adolescent Mozambican girls using food photographs ranged from an under estimate of 19% for rice to 8% for shrimp sauce[25]. Variability of estimates not only occurs with different food types but also between different individuals. Piaget and Inhelder (1974) found children's conceptual development relating to shape and volume occurs in stages[26]. Some of the cognitive processes required for the quantification of portion sizes, such as conservation (the ability to recognise that size or quantity remains the same when the appearance of the object changes) may not develop until the ages of 7 to 11 years[27]. These cognitive processes may also be less well developed in a small percentage of the adult population meaning that while for the majority of people the methods will give a good indication of the amount of food consumed; for some individuals estimates may be very inaccurate. As shown by the distribution of estimates (Fig 3) the limits of agreement for the YPFA are skewed by a small number of wildly inaccurate estimates. Timon *et al*. (2011) found older adults were less able to assess the portion size of certain foods compared with younger adults. Subsequently there is a need to include a measure of variance such as limits of agreement or standard deviation in reporting the accuracy of estimates of portion size[28].

Whilst the limits of agreement are wider than is desirable they are comparable with findings for the limits of agreement of the adult food photographs used with adults[12, 29]. Data from Nelson *et al*. (1996)[11] (analysed by Foster *et al*. (2006)[30]) reported limits of agreement from -65% to +225% with adults. Turconi *et al*. (2005) when looking at estimates made by adults and children using a series of food photographs developed for use with the Italian population reported limits of agreement for all foods tested of -114.9g to +87.8g[31].

One of the main limitations when comparing the results of our work with other studies reported in the literature is the non-standardised method of presenting results. The use of raw differences of food weights makes it difficult to interpret the data when there is amalgamation across different food types. These limitations make it difficult to draw conclusions on the relative accuracy and precision of different methods.

The accuracy and precision of estimated nutrient intakes is better than that for estimates of the portion size of individual foods. At the group level reported intakes based on the estimates of portion size using YPFA were very close to the intakes reported in the weighed food diary. The limits of agreement are relatively narrow for most nutrients and a high number of estimates are within 50% of the intake reported in the weighed diary. Energy, carbohydrate and fat are in most foods and estimates for these nutrients were generally more accurate and precise than estimates of protein, iron and vitamin C, which tend to be concentrated in a smaller range of food types.

The YPFA is practical and easy to use by both researcher and respondent and provides an alternative to the weighed intake. Further work is required to explore the impact of food type and method of presentation of foods in photographs on the accuracy of estimates of portion size of commonly consumed, yet difficult to estimate foods. Improvement in the accuracy of estimation of a small number of these foods could have a significant effect on the overall accuracy of the tool.

### Study limitations

Validation of dietary assessment methods is complicated by the lack of a true “gold standard”. Observation and recording of intakes is practicable only in certain settings, and feeding studies where the participant is provided with foods in an experimental setting may increase the awareness of the foods being consumed due to the novelty of the situation. Likewise keeping a weighed food diary will mean more attention is paid to the foods and the amounts consumed and may result in alteration to the diet to facilitate recording. Weighed intakes will also be subject to measurement error. Independent biomarkers of intake are available for a limited range of nutrients only and do not provide us with detailed information on the individual foods consumed. The YPFA was previously validated in a feeding study for a limited range of foods [22]. The purpose of the present study was to assess performance of the tool in a “real-life setting”. The weighed intake although imperfect was felt to be the best option available and where possible (i.e. in school and nursery) was supplemented by trained research staff observing and recording food intakes. The purpose of the weighed intake in this study was to provide data against which to compare the estimates of portion size. Although there are well documented problems associated with collecting weighed food intakes from participants, these mainly relate to recruiting a representative sample[4] and under-reporting of habitual intake[1]. The fact that weighing foods is the most accurate method of measuring portion size is not disputed.

## Conclusions

This comparison study found good agreement between the food atlas and weighed food diaries at the group level (i.e. population mean) but with high variability at the individual level. For children of 11 years and over agreement, with weighed food diaries, was as good as that of their parents. This is an important finding as children of this age are becoming increasingly autonomous and may consume a substantial proportion of their intake away from their parents. It is recognised that portion size estimation does result in some loss of accuracy and precision in measures of amount consumed compared with weighing of foods, however, this is offset by a reduced respondent burden and so arguably a reduced impact on the respondent’s habitual diet. The YPFA provides a method that significantly improves the estimation of weights compared with the use of adult food photographs[12] with children[32, 33]. The Young Person’s Food Atlas offers an alternative to the weighed intake in this age group.
